# Case Report: rapid progressive puberty in siblings with *MC4R* p. Arg165Gln: longitudinal follow-up and gonadotropin-releasing hormone agonist treatment

**DOI:** 10.3389/fendo.2026.1799027

**Published:** 2026-05-01

**Authors:** Hui Huang, Yu Yang, Xiangyu Xiong, Dongguang Zhang, Li Yang, Liling Xie

**Affiliations:** 1 Jiangxi Provincial Key Laboratory of Child Development and Genetics, Jiangxi Provincial Children’s Hospital (The Affiliated Children’s Hospital of Nanchang Medical College), Nanchang, China; 2 Department of Endocrinology, Metabolism and Genetics, Jiangxi Provincial Children’s Hospital (The Affiliated Children’s Hospital of Nanchang Medical College), Nanchang, China

**Keywords:** GnRH agonist, insulin resistance, lifestyle intervention, *MC4R*, monogenic obesity, rapidly progressive puberty

## Abstract

**Background:**

Pathogenic variants in melanocortin-4 receptor (*MC4R*) are the most common cause of nonsyndromic monogenic obesity. However, links between *MC4R*-related obesity and pubertal tempo, and the long-term endocrine–metabolic trajectory in East Asian children, remain insufficiently characterized. Objective: To describe the longitudinal endocrine and metabolic features and treatment response of two siblings carrying a pathogenic *MC4R* variant who developed rapidly progressive puberty with central activation.

**Case report:**

We report two Chinese siblings with severe early-onset obesity and accelerated pubertal tempo. Trio-based whole-exome sequencing with segregation analysis identified a heterozygous *MC4R* variant (NM_005912.3: p.Arg165Gln). The proband,a girl, initially presented with premature thelarche, followed by rapid progression with advanced bone age and increased growth velocity. A GnRH stimulation test demonstrated a pubertal luteinizing hormone response (peak LH 6.61 mIU/mL). GnRH agonist (GnRHa) therapy stabilized pubertal progression until discontinuation, after which spontaneous puberty resumed and menarche occurred at 13 years 6 months. At 11 years 8 months, metabolic evaluation revealed impaired glucose tolerance with hyperinsulinemia/insulin resistance and hypertriglyceridemia.Short-term metformin therapy, together with intensified lifestyle intervention, was followed by sustained weight reduction and durable glycemic normalization. At the most recent follow-up (14 years 5 months), height was 162 cm (SDS + 0.49), weight was 55 kg (SDS + 0.77), BMI was 20.96 kg/m^2^ (SDS + 0.66), and fasting blood glucose remained within the normal range. The younger brother showed concordant severe obesity with rapid pubertal progression, pubertal basal gonadotropins, and a strongly positive GnRH stimulation test (peak LH 50.82 mIU/mL; peak LH/FSH ratio 1.30). Although GnRHa was initiated relatively late, at 10 years 3 months, treatment was undertaken not only because of rapid pubertal progression, persistent bone-age advancement, and concern for compromised height potential, but also because of marked psychosocial distress and strong parental preference for active intervention. Biochemical suppression was achieved at 10 years 6 months (peak LH 0.88 mIU/mL), with stable clinical staging thereafter.

**Conclusion:**

Longitudinal follow-up of siblings carrying the pathogenic *MC4R* p. Arg165Gln variant highlights two clinical priorities in severe pediatric obesity, timely recognition and individualized management of rapidly progressive centrally activated puberty, and sustained guideline-informed surveillance and treatment of cardiometabolic comorbidities through long-term lifestyle intervention.

## Introduction

Obesity can be genetically classified into syndromic obesity, nonsyndromic monogenic obesity, and polygenic (multifactorial) obesity (1). Accumulating evidence indicates that the leptin-melanocortin pathway is a pivotal regulator of appetite and energy homeostasis and a major biological axis underlying both monogenic and polygenic forms of obesity. Within this pathway, variants in *MC4R* are identified in 3–5% of individuals with severe, early-onset obesity in many cohorts (1–3).

*MC4R* deficiency (OMIM #618406) typically manifests as early-onset severe obesity with hyperphagia, increased lean mass, accelerated linear growth, and hyperinsulinemia that is disproportionate to the degree of obesity (4). However, evidence from Chinese pediatric cohorts remains limited, with only four published case reports of pathogenic *MC4R* variants (5–8). Moreover, rapidly progressive puberty has been rarely reported or is not well characterized in individuals carrying pathogenic *MC4R* variants, and longitudinal data integrating growth trajectories, pubertal tempo, and evolving metabolic complications are scarce—particularly in monogenic obesity accompanied by rapidly progressive puberty.

Here, we report a Chinese Han family carrying the pathogenic *MC4R* c.494G>A (p. Arg165Gln; R165Q) variant, in which two siblings presented with early-onset obesity and rapidly progressive puberty. We provide a six-year follow-up documenting pubertal suppression with a gonadotropin-releasing hormone agonist (GnRHa) and subsequent metabolic management. By delineating phenotype evolution and treatment response over time, this case report expands the clinical spectrum of *MC4R*-related obesity and may improve recognition and management of rapidly progressive puberty in similar presentations.

## Case 1 (proband)

An 8-year-7-month-old girl was referred to the Endocrinology Clinic because of progressive bilateral breast enlargement over the preceding 9 months. At 7 years and 10 months of age, she developed painless bilateral thelarche and sex hormone levels, bone age, and pelvic ultrasonography did not suggest pubertal acceleration; therefore, she was considered to have premature thelarche and was followed up regularly. Over the subsequent 9 months, breast enlargement progressed and was accompanied by tenderness, and her recent height velocity was 8 cm/year. Review of systems was negative for headaches, visual disturbances, vomiting, seizures, acne, body odor, vaginal discharge, or vaginal bleeding. There was no known exposure to exogenous sex steroids, including estrogen-containing creams or supplements.

She was born at term via spontaneous vaginal delivery (birth weight 2.95 kg; length 50 cm). She was the first child of non-consanguineous parents, and the family reported no history of inherited disorders or miscarriage. Her growth and developmental milestones were reportedly age-appropriate. She had a good appetite since early childhood with a stocky habitus and reported no snoring or other sleep-related symptoms at presentation. Maternal age at menarche was 13 years, while paternal pubertal timing was unavailable.

**Figure 1:**
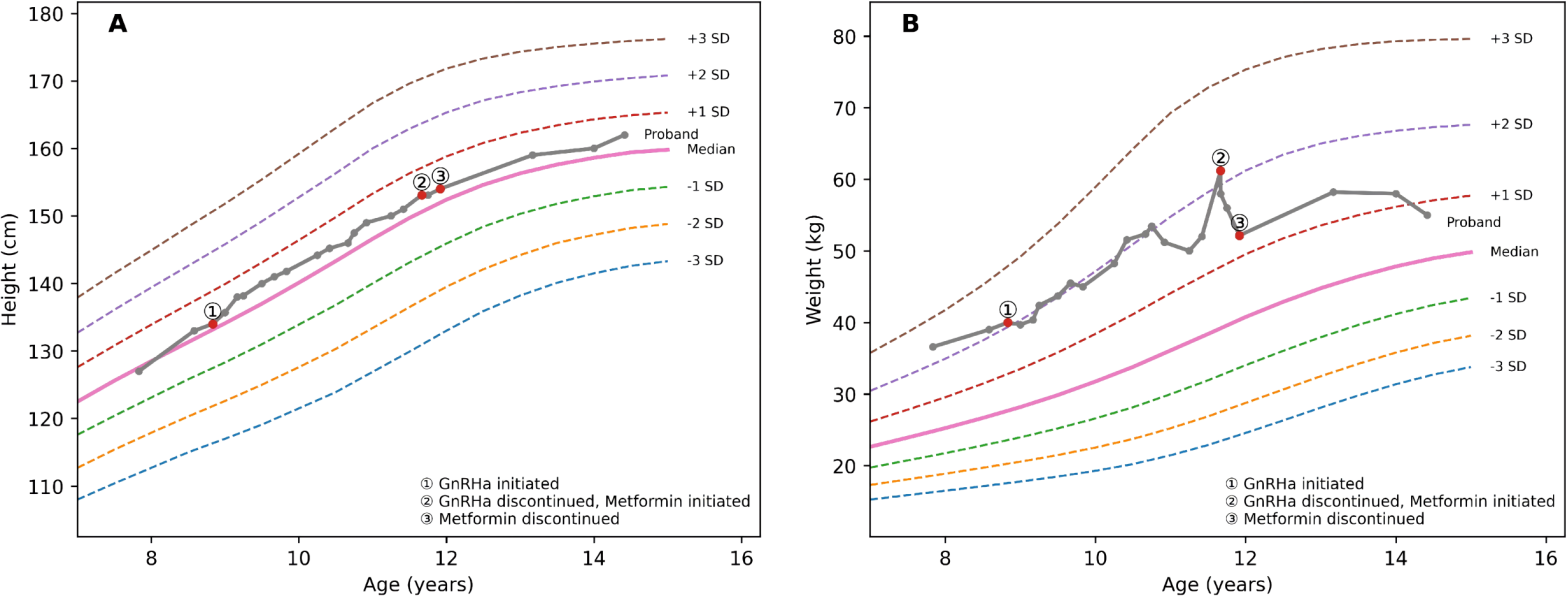
Growth curves of the proband carrying the MC4R c.494G>A (p.R165Q) variant over 6-year follow-up. Curves display longitudinal changes in height **(A)** and weight **(B)** plotted against age- and sex-matched reference data from the Chinese Children’s Growth Standards (2009) (9, 10). Solid lines represent the population median and the proband’s measurements, and dashed lines indicate ±1, ± 2, and ±3 standard deviations (SD). Treatment milestones are marked: ① GnRHa initiated; ② GnRHa discontinued and metformin initiated; ③ metformin discontinued.

On examination, blood pressure was 105/65 mmHg (below the 90th percentile for age, sex, and height). Height was 133 cm (SDS + 0.19), weight 39 kg (SDS + 2.11), waist circumference 68 cm (waist-to-height ratio 0.51), and body mass index (BMI) 22.1 kg/m^2^ (SDS 2.87) (**Figures 1A, B**; **Table 1**). No acanthosis nigricans, café-au-lait macules, cutaneous neurofibromas, or other neurocutaneous stigmata were observed. There were no Cushingoid features or goiter. Breast development was Tanner stage B2; palpation confirmed glandular breast tissue with tenderness. Areolar hyperpigmentation was absent. Pubic hair was Tanner stage PH1. Cardiopulmonary and abdominal examinations were otherwise unremarkable.

**Table 1:** Longitudinal endocrine, metabolic, and imaging assessments across key clinical milestones in the proband.

Variable	Reference	7y10m	8y7m	9y	9y8m	10y3m	10y11m	11y5m	11y8m ②	11y11m③	13y2m
Treatment	-	Pre	Pre	GnRHa (start 8y9m①)	GnRHa	GnRHa depot	GnRHa depot	GnRHa final	Stop GnRHa + Metformin②	Stop Metformin ③	Off
Growth parameters											
Height (cm)	–	127	133	135.7	141	144.2	149	151	153.1	154	159
Height SDS	–	-0.1	0.19	0.27	0.4	0.37	0.43	0.26	0.37	0.32	0.37
Weight (kg)	–	36.6	39	39.7	45.5	48.25	51.2	52	61.2	52.15	58.2
Weight SDS	–	2.37	2.11	1.91	2.02	1.9	1.71	1.49	2.13	1.26	1.36
BMI	–	22.7	22	21.6	22.9	23.2	23.1	22.8	26.1	22	23
BMI SDS	–	3.67	2.86	2.45	2.63	2.47	2.23	1.95	2.74	1.55	1.50
Pubertal staging											
Breast stage (B)	–	B2	B2	B2	B2	B2	B2	B2	B3	B3	B4
Pubic hair (PH)	–	PH1	PH1	PH1	PH1	PH1	PH1	PH1	PH1	PH2	PH4
Reproductive hormones (basal)											
FSH (mIU/mL)	1-4.2	1.11	2.6	0.8	3.34	2.54	2.43	2.19	–	–	1.47
LH (mIU/mL)	0.1-5.4	<0.08	0.09	0	0.09	0	<0.08	<0.08	–	–	0.38
Estradiol (pg/mL)	4.9-19.9	<11.8	**33.53**	**40.9**	**32.44**	**25.08**	**35.47**	**37.23**	**-**	**-**	**54.19**
GnRH stimulation test											
Peak LH (mIU/mL)	–	–	6.61	0	–	–	–	–	–	–	–
Peak FSH (mIU/mL)	–	–	13.70	0.71	–	–	–	–	–	–	–
Peak LH/FSH	–	–	0.48	0	–	–	–	–	–	–	–
Lipid profile											
TC (mmol/L)	3.6-6.7	–	4.62	4.86	4.58	4.14	4.53	5.29	–	4.45	–
TG (mmol/L)	0.4-1.8	–	**2.1**	**2.88**	**4.34**	**2.34**	**4.78**	**6.64**	–	**2.1**	–
HDL-C (mmol/L)	0.72–1.71	–	1.24	1.11	1.05	1.07	1.11	1.37	–	1.05	–
LDL-C (mmol/L)	2.07–3.1	–	2.57	2.68	2.3	2.39	2.04	2.64	–	2.62	–
Non-HDL-C (mmol/L)*	–	–	3.38	**3.75**	3.53	3.07	3.42	**3.92**	–	3.4	–
FFA (mmol/L)	0.4–0.9	–	0.74	0.75	0.54	0.42	<0.2	0.31	–	0.2	–
Glucose metabolism											
FBG (mmol/L)	3.89-6.11	–	4.28	4.17	5.74	5.98	**7.84**	**8.2**	5.02	4.76	–
FINS (µU/mL)	4.03–23.46	–	9.72	–	–	–	–	–	22.95	22.45	–
HOMA-IR*	–	–	1.85	–	–	–	–	–	**5.12**	**4.75**	–
HbA1c (%)	3.8–5.8	–	–	–	–	–	–	–	3.8	4.7	–
2 h glucose (mmol/L)	–	–	–	–	–	–	–	–	8.03	7.58	–
2 h insulin (µU/mL)	–	–	–	–	–	–	–	–	119.6	156.3	–
2 h C-peptide (µU/mL)									16.65	13.64	
Liver enzymes and uric acid											
ALT (U/L)	7–30	–	19	28	19	14	22	**41.4**	–	18	–
AST (U/L)	14–44	–	30	42	23	20	19	26.5	–	22	–
GGT (U/L)	5–19	–	13	14	19	17	**23**	**30.8**	–	**20**	–
ALP (U/L)	146–500	–	341	299	379	344	431	328.8	–	292	–
Uric acid (µmol/L)	123–430	–	308	367.6	358.7	335.2	296.4	**546**	–	379.4	–
Bone age and pelvic ultrasonography											
Bone age (BA)	–	8y6m	10y6m	–	–	11y5m	11y5m	11y8m	–	–	13y5m
BA–CA		+8m	**+1y11m**			**+1y2m**	+6m	+3m	–	–	+3m
Uterine length (cm)	–	2.9	3.2	3.2	3.2	3.3	3.3	3.7	–	–	5.6
Left ovary (ml)	–	0.854	1.904	1.641	1.551	1.620	1.728	1.636	–	–	3.985
Right ovary (ml)	–	1.105	2.107	1.727	1.636	1.751	1.656	1.647	–	–	3.949

–, not assessed. Reference intervals are age- and sex-specific. Values outside the reference range are in bold. ① GnRHa initiated at 8y9m. ② GnRHa discontinued and metformin initiated at 11y8m. ③ Metformin discontinued at 11y11m. * HOMA-IR = fasting insulin × fasting glucose/22.5 (reference ≤3.0) (11). ※ Uterine length >3.2 cm has been suggested as supportive of pubertal uterine maturation. Bone age was assessed using the Greulich–Pyle atlas. BA–CA was calculated as bone age minus chronological age; positive values indicate advanced bone age.

Baseline gonadotropins and sex steroids were measured by chemiluminescent immunoassay using an automated chemiluminescence analyzer (DxI 800, Beckman Coulter, USA) with the corresponding reagent kits. and additional biochemical indices were assessed using standard automated platforms; results were interpreted using assay- and age-/sex-specific reference ranges (**Table 1**). Basal FSH was 2.6 mIU/mL and LH 0.09 mIU/mL; estradiol was 33.53 pg/mL. GnRH stimulation testing (intravenous gonadorelin) with sampling at 0 and 60 min showed a peak LH of 6.61 mIU/mL and a peak FSH of 13.7 mIU/mL (peak LH/FSH ratio 0.48). Although the peak LH/FSH ratio was <0.6, the stimulated LH exceeded the commonly used cutoff of 5 IU/L (equivalent to 5 mIU/mL) and, together with progressive breast development, increased height velocity, and advanced bone age, supported central pubertal activation. A fasting metabolic evaluation showed hypertriglyceridemia (TG 2.1 mmol/L; reference 0.4-1.8), whereas total cholesterol, HDL-C, LDL-C, and free fatty acids were within the reference ranges. Fasting glucose was 4.28 mmol/L (reference 3.89-6.11) with fasting insulin 9.72 μU/mL (reference 4.03-23.46), yielding a HOMA-IR of 1.85. Other endocrine evaluations (including thyroid axis and adrenal steroids), liver and kidney function, uric acid, electrolytes, complete blood count, and hepatitis B serology were within normal ranges (**Table 1**).

Bone age, assessed using the Greulich-Pyle atlas, was 10.6 years, advanced by 1.9 years relative to her chronological age of 8.7 years. Mid-parental target height was 159.3 cm (target range 150.8-167.8 cm). Abdominal (hepatic) ultrasonography was normal with no evidence of nonalcoholic fatty liver disease (NAFLD). Pelvic ultrasonography showed a uterine length of 3.2 cm. Brain MRI was obtained because of the progressive pubertal course and advanced bone age and showed no abnormalities.

**Figure 2:**
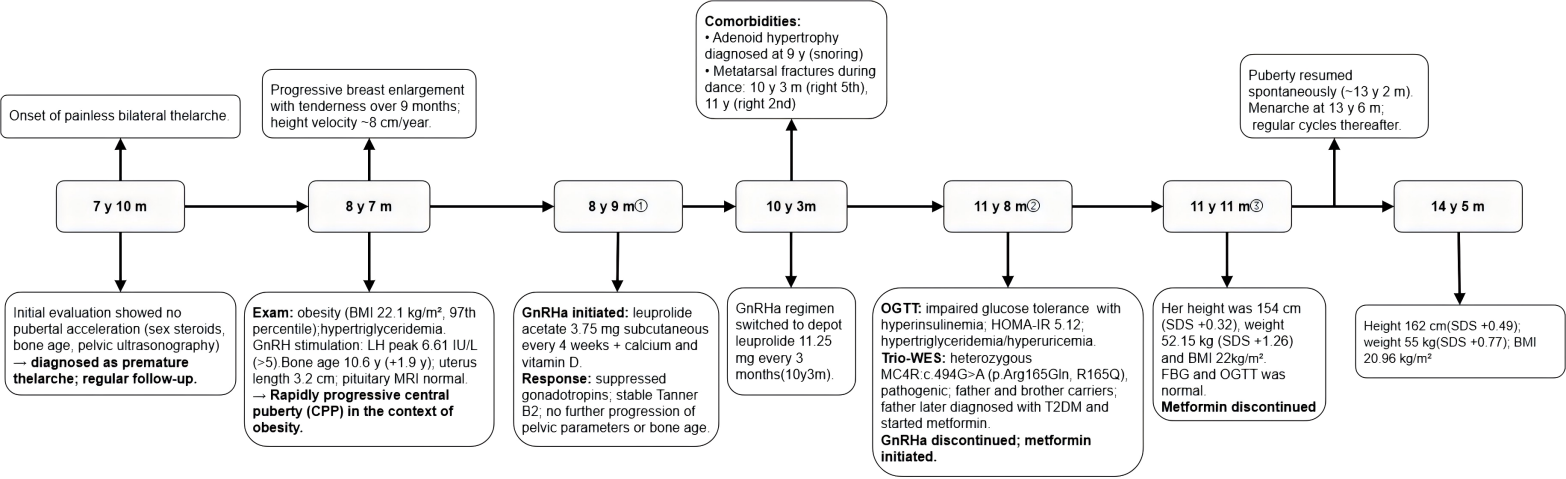
Diagnosis and treatment timeline of the proband (Case 1). The horizontal axis indicates the patient’s age at key clinical milestones. All abbreviations are listed in the Abbreviations section.

The proband was assessed as having rapidly progressive puberty with evidence of central activation, evolving from initial premature thelarche, in the context of obesity and hypertriglyceridemia. The family initially declined obesity-related evaluation. Given rapid pubertal progression with persistently advanced bone age, concerns regarding compromised adult height potential, and psychosocial distress related to early pubertal changes, the family agreed to puberty-suppressing therapy (**Figure 2**).

**Figure 3:**
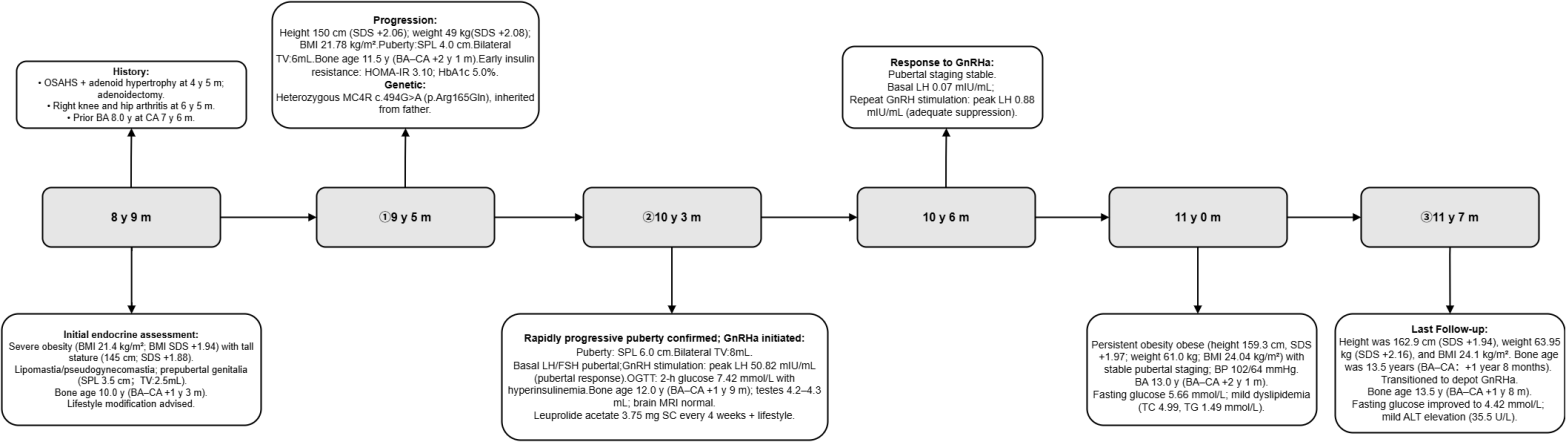
*MC4R* c.494G>A (p.R165Q) variant: family pedigree, Sanger sequencing validation, conservation analysis, and 3D structural model. **(A)** Pedigree showing the proband (arrow) and family members carrying the *MC4R* c.494G>A (p.R165Q) variant. **(B)** Sanger sequencing chromatograms confirm heterozygous c.494G>A in the proband, father, and younger brother. **(C)** Cross-species conservation analysis of the affected residue (Arg165) and a 3D structural model illustrating the Arg165Gln substitution in the third intracellular loop, a region implicated in G-protein coupling and signal transduction. Prior functional studies suggest that this variant reduces ligand responsiveness and downstream signaling.

① 8 years and 9 months: GnRHa initiated. GnRHa therapy was started with leuprolide acetate (3.75 mg subcutaneously every 4 weeks). During GnRHa treatment, growth velocity was 7.0 cm/year in year 1, 5.2 cm/year in year 2, and 5.8 cm/year in year 3. Treatment efficacy was supported by serial gonadotropin measurements showing suppressed basal LH and FSH, and by repeat GnRH stimulation testing at 9 years-old, demonstrating a suppressed gonadotropin response. Clinically, breast Tanner stage remained B2, and uterine/ovarian parameters and bone age showed no further progression during GnRHa therapy (**Table 1**).

At 9 years of age, adenoid hypertrophy was diagnosed by an otolaryngologist; there were no clinically significant symptoms of sleep-disordered breathing. She sustained fractures of the right fifth metatarsal at 10 years and 3 months and the right second metatarsal at 11 years during dance activities; both were treated with plaster immobilization and healed uneventfully. At 10 years and 3 months, because of fractures and the COVID-19 pandemic, the regimen was switched to a long-acting depot formulation (leuprolide acetate 11.25 mg every 3 months) to reduce the frequency of hospital visits. The final dose was administered at 11 years and 5 months, when breast development remained at Tanner stage B2 and bone age was approximately 11 years and 8 months.

② 11 years and 8 months: GnRHa discontinued; metformin initiated. At 11 years and 8 months, her height was 153.1 cm (SDS + 0.38), weight 61.2 kg (SDS + 2.13), waist circumference 80 cm (waist-to-height ratio 0.52), and BMI 26.1 kg/m^2^(SDS + 2.74). Given previously documented fasting hyperglycemia at earlier visits (**Table 1**), the family consented to a comprehensive obesity evaluation. Oral glucose tolerance testing demonstrated impaired glucose tolerance (0 min 5.02 mmol/L; 120 min 8.03 mmol/L), accompanied by hyperinsulinemia (0 min insulin 22.95 μU/mL; 120 min insulin 119.6 μU/mL) and insulin resistance (HOMA-IR 5.12), together with persistent hypertriglyceridemia and hyperuricemia (**Table 1**). Abdominal (hepatic) ultrasonography was normal with no evidence of nonalcoholic fatty liver disease (NAFLD). Metformin was initiated at a dose of 0.25 g three times daily, alongside intensified dietary and physical-activity counseling.

At the same visit, with informed consent from the proband and her family, trio-based whole-exome sequencing (WES; proband, parents, and younger brother) identified a heterozygous *MC4R* missense variant, NM_005912.3: c.494G>A, p.(Arg165Gln) (R165Q). The variant was classified as pathogenic according to ACMG criteria (PM1, PM5, PP3, and PS4 [very strong]). Sanger sequencing confirmed the variant in the proband, father, and younger brother, while the mother tested negative (**Figure 3**). The father (38 years) was 169 cm tall (SDS -0.61) and weighed 80 kg (BMI 28.0 kg/m^2^). After genetic confirmation, he underwent evaluation at an external hospital and was found to have a fasting plasma glucose concentration >11.1 mmol/L, leading to a diagnosis of type 2 diabetes mellitus; metformin was initiated with regular glucose monitoring. The paternal grandmother reportedly had obesity and diabetes mellitus; further details were unavailable. The mother (36 years) was 162.5 cm tall (SDS + 0.35) and weighed 50 kg (BMI 18.9 kg/m^2^).

**Figure 4:**
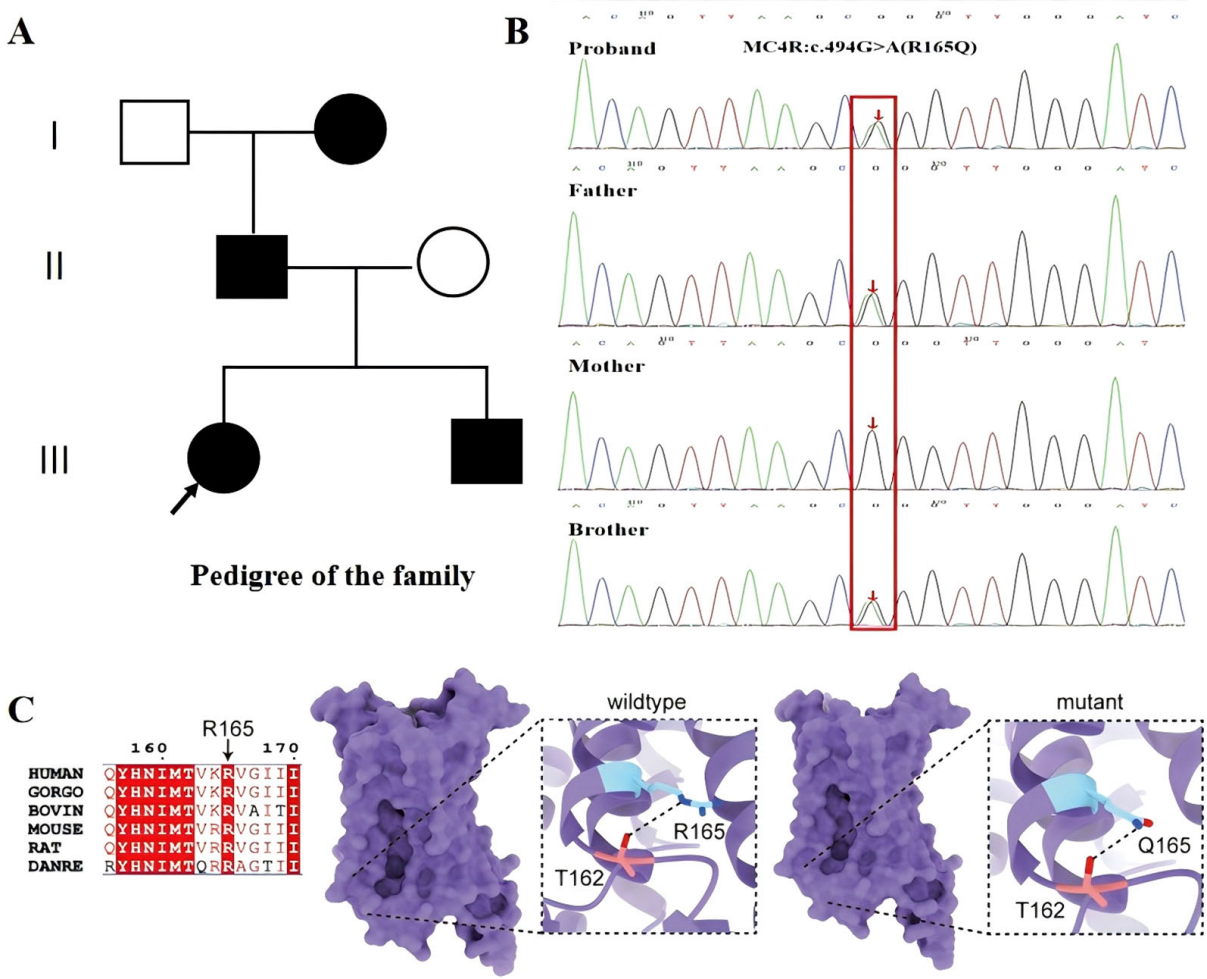
Diagnosis and treatment timeline of the younger brother (Case 2). The timeline summarizes key milestones from past history to endocrine evaluation, rapid pubertal progression, confirmation of central pubertal activation, initiation of GnRHa, biochemical suppression during treatment, metabolic follow-up, transition to depot formulation, and genetic confirmation of the familial MC4R c.494G>A (p.Arg165Gln) variant. Ages are shown as years (y) and months (m). Bone age was assessed by the Greulich–Pyle method, and BA–CA indicates bone age advancement. All abbreviations are listed in the Abbreviations section.

③ 11 years and 11 months: metformin discontinued. At 11 years and 11 months, her height was 154 cm (SDS + 0.32), weight 52.15 kg (SDS + 1.26), and BMI 22.0 kg/m^2^(SDS + 1.55). Over the preceding three months, she lost 10 kg; triglycerides decreased to 2.1 mmol/L, uric acid normalized, and OGTT showed normalization of glucose tolerance (120 min glucose 7.58 mmol/L), although hyperinsulinemia and insulin resistance persisted (**Table 1**). Following the decrease in body weight and improvement in laboratory parameters, the parents requested that metformin be discontinued. Metformin was therefore stopped, and diet and lifestyle intervention were continued. At 13 years and 2 months, her height was 159 cm (SDS + 0.37), and spontaneous pubertal progression resumed. Menarche occurred at 13 years and 6 months, followed by regular cycles. At the most recent follow-up (14 years and 5 months), height was 162 cm (SDS + 0.49), weight 55 kg (SDS + 0.77), BMI 20.96 kg/m^2^(SDS + 0.66), and fasting blood glucose remained within the normal range.

## Case 2 (younger brother)

An 8-year-9-month-old boy, the proband’s younger brother, was born at 37 weeks’ gestation via spontaneous vaginal delivery (birth weight 3.6 kg). At 4 years 5 months, he was diagnosed with obstructive sleep apnea–hypopnea syndrome and adenoid hypertrophy and underwent adenoidectomy. Prior records documented that a bone age assessment was performed at 7 years 6 months, showing a bone age of 8.0 years, with no significant advancement relative to chronological age.

At 8 years 9 months of age, the patient presented to the Endocrinology Clinic for evaluation of growth and development because of bilateral breast enlargement for 6 months. The patient reported increased appetite. No snoring, apnea, daytime somnolence, or other sleep-related symptoms were reported. At the initial endocrine assessment (8 years 9 months), his height was 145 cm (SDS + 1.88), weight 45 kg (SDS + 2.09), waist circumference 78 cm (waist-to-height ratio 0.53), and BMI 21.4 kg/m^2^ (SDS +1.94), **Table 2**. Blood pressure was within the normal range. Examination showed marked adiposity with obesity-related breast enlargement (lipomastia) and prepubertal genital development (Tanner G1) without pubic hair (PH1). Stretched penile length was 3.5 cm. Bilateral testicular volume(TV) was 2.5 mL, assessed using a Prader orchidometer.Bone age, assessed by the Greulich–Pyle method was 10.0 years (BA–CA:+1 year 3 months). Nutritional counseling and lifestyle modification were recommended (**Figure 4**).

**Table 2:** Longitudinal endocrine, metabolic, and imaging assessments across key clinical milestones in CASE 2 (Younger brother).

Variable	Reference	8y9m	9y5m	10y3m	10y6m	11y	11y7m
Treatment	-	Pre	Pre	GnRHa start	GnRHa	GnRHa	GnRHa depot
Growth parameters							
Height (cm)		145	150	153.5	155.5	159.3	162.9
Height SDS		1.88	2.06	2.0	1.97	1.97	1.94
Weight (kg)		45	49	53	53.35	61	63.95
Weight SDS		2.09	2.08	1.96	1.9	2.19	2.16
BMI		21.4	21.78	22.49	22.06	24.04	24.1
BMI SDS		1.94	1.84	1.77	1.61	1.97	1.84
Pubertal staging							
Genital stage (G)	–	G1	G2	G2	G2	G2	G2
Penile (cm)	–	3.5	4	6	6	6	6
TV by Prader orchidometer, left (mL)	–	2.5	6	8	8	8	8
TV by Prader orchidometer, Right (mL)	–	2.5	6	8	8	8	8
Pubic hair (PH)	–	PH1	PH2	PH2	PH2	PH2	PH2
Reproductive hormones (basal)							
FSH (mIU/mL)	1.8-3.2	–	–	9.06	0.59	0.46	0.97
LH (mIU/mL)	0.02-0.3	–	–	1.15	0.07	0.30	0.13
Testosterone (ng/dL)	7–29.44	–	–	22.51	14.01	17.67	16.44
GnRH stimulation test							
Peak LH (mIU/mL)	–	–	–	50.82	0.88	–	–
Peak FSH (mIU/mL)	–	–	–	39.12	0.62	–	–
Peak LH/FSH	–	–	–	1.30	–	–	–
Lipid profile							
TC (mmol/L)	3.12-4.92	–	4.27	4.2	5.22	4.99	–
TG (mmol/L)	≤2.3	–	0.9	1.22	2.08	1.49	–
HDL-C (mmol/L)	0.72–1.71	–	1.55	1.16	1.34	1.48	–
LDL-C (mmol/L)	2.07-3.1	–	2.27	2.36	2.79	2.86	–
Non-HDL-C (mmol/L)	–	–	2.72	3.04	3.88	3.51	–
FFA (mmol/L)	0.4–0.9	–	0.51	0.4	0.19	0.42	–
Glucose metabolism							
FBG (mmol/L)	3.89-6.11	–	4.67	5.89	5.78	5.66	4.42
FINS (µU/mL)	4.03–23.46	–	15	11.72	–	–	–
C-peptide (µU/mL)	0.3-3.73	–	2.45	2.48	–	–	–
HOMA-IR*	–	–	3.1	3.07	-	–	–
HbA1c (%)	3.8–5.8	–	5.0	4.8	–	–	–
2 h glucose (mmol/L)	–	–	–	7.42	–	–	–
2 h insulin (µU/mL)	–	–	–	58.82	–	–	–
2 h C-peptide (µU/mL)	–	–	–	9.71	–	–	–
Liver enzymes and uric acid							
ALT (U/L)	7–30	–	20.07	21	28	30	35.5
AST (U/L)	14–44	–	19.65	21	25	25	21.98
GGT (U/L)	5–19	–	18.51	18	19	20	15
ALP (U/L)	146–500	–	330.85	429	290	362	258.18
Uric acid (µmol/L)	123–430	–	318.96	328.8	308	339.7	352.9
Bone age and pelvic ultrasonography							
Bone age (BA)	–	10y	11.5y	12y	–	13y	13.5y
BA–CA	–	+1y3m	+2y1m	+1y9m	–	+2y1m	+1y8m
Left Testicular volume (US, mL)	–	–	–	4.2	–	–	4.3
Right testicular volume (US, mL)	–	–	–	4.3	–	–	4.4

-, not assessed. Reference intervals are age- and sex-specific. Values outside the reference range are in bold. * HOMA-IR = fasting insulin × fasting glucose/22.5 (reference ≤3.0) (11). Bone age was assessed using the Greulich–Pyle atlas. BA–CA was calculated as bone age minus chronological age; positive values indicate advanced bone age.

① At 9 years 5 months, the patient was confirmed to carry the familial heterozygous *MC4R* c.494G>A (p. Arg165Gln) variant following trio-WES, and he returned to the clinic for re-evaluation of growth and pubertal development. Anthropometric indices remained elevated: height 150 cm (SDS + 2.06), weight 49 kg (SDS + 2.08), and BMI 21.78 kg/m^2^ (SDS + 1.84). Between 8 years 9 months and 9 years 5 months, height increased from 145.0 to 150.0 cm, corresponding to a growth velocity of approximately 7.5 cm/year. Pubertal findings progressed to Tanner G2 with pubic hair stage PH2. Stretched penile length was 4.0 cm. Bilateral TV was estimated at 6 mL by Prader orchidometer.Bone age advanced to 11.5 years (BA–CA +2 years 1 month). Metabolic screening showed early insulin resistance (FBG 4.67 mmol/L, fasting insulin 15 µU/mL; HOMA-IR 3.10) with HbA1c 5.0% and no abnormalities in liver enzymes or lipid profile. Hepatic ultrasonography was normal with no evidence of NAFLD. Nutritional counseling and lifestyle modification were recommended and maintained throughout follow-up.

② At 10 years 3 months, genital staging remained Tanner G2 with pubic hair stage PH2. Stretched penile length was 6.0 cm, and bilateral TV was clinically 8 mL by Prader orchidometer. Height was 153.5 cm (SDS + 2.00), weight 53 kg (SDS + 1.96), and BMI 22.49 kg/m^2^(SDS + 1.77). Between 9 years 5 months and 10 years 3 months, height increased from 150.0 to 153.5cm, corresponding to a growth velocity of approximately 4.2 cm/year. Basal reproductive hormones were in the pubertal range (LH 1.15 mIU/mL, FSH 9.06 mIU/mL) with testosterone 22.51 ng/dL. A GnRH stimulation test showed a pubertal response (peak LH 50.82 mIU/mL; peak FSH 39.12 mIU/mL; peak LH/FSH 1.30), confirming central activation of the hypothalamic–pituitary–gonadal axis. Oral glucose tolerance testing showed normal 2-hour glucose (7.42 mmol/L) but hyperinsulinemia (2-hour insulin 58.82 μU/mL) and insulin resistance (FBG 5.89 mmol/L; fasting insulin 11.72 μU/mL; HOMA-IR 3.07). BA was 12.0 years (BA–CA:+1 year 9 months). Testicular ultrasonography showed volumes of 4.2 mL (left) and 4.3 mL (right). Brain MRI was performed as part of the etiologic evaluation and was normal.

Following recognition of the familial genetic background, the parents became more attentive to the younger brother’s pubertal progression. Although GnRHa treatment had been discussed earlier, the family initially preferred close observation. During short-interval follow-up, pubertal progression became more evident, and the patient developed increasing distress related to these changes. After comprehensive reassessment at 10 years 3 months, the parents strongly preferred active treatment. GnRHa was therefore initiated on the basis of progressive central puberty, persistent bone-age advancement, concern for compromised height potential, and psychosocial burden, rather than an expectation of substantial adult-height gain alone. Treatment was started with leuprolide acetate 3.75 mg subcutaneously every 4 weeks, together with continued nutritional counseling and lifestyle intervention.

At 10 years 6 months, 3 months after GnRHa initiation, anthropometric indices remained elevated (height 155.5 cm, SDS + 1.97; weight 53.35 kg, SDS + 1.90; BMI 22.06 kg/m^2^, SDS + 1.61), Pubertal findings remained stable, with no further progression during treatment. Basal gonadotropins were low (LH 0.07 mIU/mL, FSH 0.59 mIU/mL), with testosterone 14.01 ng/dL. Repeat GnRH stimulation testing with sampling at 0 and 60 minutes demonstrated biochemical suppression (peak LH 0.88 mIU/mL; peak FSH 0.62 mIU/mL), consistent with adequate hypothalamic-pituitary-gonadal axis suppression during treatment.

At 11 years, he remained obese, with height 159.3 cm (SDS + 1.97), weight 61.0 kg (SDS + 2.19), and BMI 24.04 kg/m^2^ (SDS + 1.97). Pubertal findings remained stable, with no further progression. Blood pressure was within normal range throughout follow-up. Bone age was 13.0 years (BA–CA +2 years 1 month). Metabolic monitoring showed fasting glucose of 5.66 mmol/L and mild dyslipidemia, with total cholesterol of 4.99 mmol/L and triglycerides of 1.49 mmol/L.

③ At 11 years 7 months, he transitioned to a depot GnRHa formulation. Pubertal findings remained stable, with no further progression. Bilateral testicular volume remained approximately 8 mL by Prader orchidometer, and testicular ultrasonography showed volumes of 4.3 mL on the left and 4.4 mL on the right. Height was 162.9 cm (SDS + 1.94), weight 63.95 kg (SDS + 2.16), and BMI 24.1 kg/m^2^ (SDS + 1.84). Bone age was 13.5 years (BA–CA:+1 year 8 months). Fasting glucose improved to 4.42 mmol/L; liver enzymes showed a mild ALT elevation (35.5 U/L). During follow-up, hepatic ultrasonography remained normal, with no evidence of NAFLD.

## Patient perspective

The family’s main concerns were the child’s rapid pubertal progression, possible compromise of adult height, and increasing metabolic risks associated with severe obesity. Influenced in part by traditional beliefs, including the perception that a heavier child is healthier, they initially paid limited attention to early obesity management. As pubertal development progressed rapidly, they became increasingly concerned about the potential social and psychological pressure of early maturation on the child. After counseling, the family accepted puberty-suppressing therapy and reported good adherence, finding the stabilization of pubertal changes and growth reassuring. When metabolic abnormalities emerged, they accepted early treatment with metformin; however, after improvement, they hoped to discontinue medication and rely primarily on diet and exercise, reflecting a preference for lifestyle-based management whenever feasible. Following confirmation of the genetic diagnosis, the parents reported a clearer understanding of the biological basis of obesity, greater awareness of weight-related health risks, and stronger commitment to sustained lifestyle intervention. They also considered the genetic findings helpful for guiding long-term follow-up and improving risk awareness among relatives; in addition, the father became more aware of his own weight and glycemic concerns and subsequently sought standardized care in an adult endocrinology/metabolic clinic.

## Discussion

*MC4R* is a core component of the hypothalamic leptin–melanocortin pathway (12), and pathogenic variants represent the most common cause of nonsyndromic monogenic obesity (13). Typical clinical features include early and severe weight gain with increased appetite, and metabolic abnormalities (particularly hyperinsulinemia and dyslipidemia) may emerge early in life (13, 14). Hyperinsulinemia is reported more frequently in children than adults (11, supporting the concept that metabolic signaling may be particularly pronounced during childhood.

Larsen et al. (15) conducted a cohort study of 750 Danish males with adolescent-onset obesity and identified 19 (2.5%) carriers of pathogenic *MC4R* variants, including 6 (0.8%) with the c.494G>A (p. Arg165Gln; R165Q) substitution. Logan et al. (2) reported the highest overall frequency of *MC4R* variants in a South African cohort (9.89%, 8/297); only one individual harbored c.494G>A and had a normal BMI, consistent with incomplete penetrance of R165Q. Population-based and clinical sequencing studies across diverse ancestries—including African and Chinese cohorts—highlight variability in *MC4R* allele frequency and phenotypic expressivity (2, 3). Reduced energy expenditure has also been described in carriers of specific functional variants, such as R165Q (16). Consistent with mechanistic work showing that *MC4R* mutations can disrupt receptor signaling and/or cell-surface expression (17, 18).

The relationship between *MC4R*-related obesity and pubertal tempo remains incompletely defined. In aggregated clinical cohorts of *MC4R* mutation carriers, advanced bone age has been reported, whereas no consistent alteration in the overall course of puberty and fertility has been observed at the population level (13). Evidence from teleost models suggests that melanocortin signaling can, under certain evolutionary contexts, directly gate pubertal timing: in Xiphophorus swordtail fish, naturally occurring *mc4r* alleles influence male puberty onset and body-size polymorphism through receptor dimerization and dominant-negative interactions (19). However, this effect may not be conserved across species, as *mc4r* loss in medaka primarily affects embryonic development and growth with little or no impact on pubertal timing (20), highlighting potential lineage-specific mechanisms and redundancy in reproductive control. More broadly, obesity has long been associated with altered pubertal timing, although the relationship is complex and likely multifactorial. In our siblings, severe early-onset obesity preceded rapid pubertal progression, raising the possibility that obesity-related metabolic signals, including insulin, leptin, and IGF-related pathways, may have provided permissive or accelerating input to the hypothalamic–pituitary–gonadal axis and skeletal maturation. At the same time, shared upstream etiologic mechanisms may also contribute.

In humans, common variants near *MC4R* (e.g., rs17782313) show robust associations with obesity, yet their relationship with puberty timing appears weak or inconsistent (21). By contrast, a recent study reported significant associations between *MC4R* variants (rs571312 and rs12970134) precocious puberty risk in Chinese girls with obesity, suggesting that *MC4R*-related genetic background may contribute to pubertal timing in specific populations or metabolic contexts (27). Importantly, the broader melanocortin network provides a plausible biological bridge between energy status, growth, and sexual maturation: loss-of-function mutations in *MC3R* are associated with delayed puberty and altered childhood growth/IGF-1 axis, underscoring that melanocortin signaling modulates developmental tempo in humans (22). Mechanistically, leptin’s permissive effects on puberty are mediated in part through arcuate AgRP neurons—an endogenous antagonist of *MC4R*—where experimental studies show that AgRP signaling imposes inhibitory tone on pubertal activation and that *MC4R* activation can engage downstream TAC2/KNDy pathways relevant to GnRH regulation (23). These findings support a plausible mechanistic link between MC4R-related obesity and pubertal tempo. As a key component of the leptin–melanocortin pathway, MC4R integrates energy balance with hypothalamic neuroendocrine signaling. Altered melanocortin tone, together with obesity-related hyperinsulinemia and leptin/IGF-related signals, may influence skeletal maturation and the timing of pubertal activation, although a direct causal role of MC4R dysfunction in accelerated puberty remains unproven.

In the clinical setting, a parsimonious interpretation is that severe early-onset obesity with early insulin resistance provides permissive or accelerating signals to the hypothalamic–pituitary–gonadal axis and skeletal maturation (via insulin, leptin, and IGF-related pathways), while altered melanocortin tone (POMC–*MC4R* vs AgRP antagonism) may modulate the central “gate” controlling pubertal tempo. Regardless of the underlying mechanism, these cases suggest that rapidly progressive puberty in children with severe early-onset obesity and metabolic abnormalities should prompt consideration of monogenic obesity. Careful longitudinal assessment of growth, bone age, and pubertal tempo is essential, particularly when progression may compromise adult height potential and adversely affect psychological well-being or psychosocial adjustment.

Children with obesity and early pubertal development require a comprehensive evaluation. In the proband, GnRH stimulation showed a pubertal peak LH but a borderline peak LH/FSH ratio; given objective pubertal progression, increased growth velocity, and advanced bone age, the overall pattern supported central activation. In children with obesity, stimulated LH responses may be blunted, making strict biochemical thresholds less reliable in isolation and emphasizing the value of integrating clinical tempo, growth, and skeletal maturation (24).

In the younger brother, central activation was supported by pubertal basal gonadotropins, a strongly positive GnRH stimulation test, and testicular volumes exceeding 4 mL. Brain MRI was performed as part of the etiologic evaluation and was normal. Although boys with central precocious puberty (CPP) have traditionally been considered at increased risk for underlying CNS lesions, more recent studies suggest that the prevalence of clinically significant organic pathology may be lower than historically reported (25), particularly in otherwise healthy boys without neurological symptoms. In this patient, the strong family history, the confirmed genetic background of obesity, and the obesity-related metabolic context were relatively reassuring; nevertheless, MRI was obtained to complete the diagnostic evaluation.

From a therapeutic perspective, GnRHa was initiated because pubertal changes progressed rapidly with advanced bone age, raising concern for loss of predicted adult height and potential psychosocial burden (24). Treatment achieved biochemical suppression and clinical stabilization in both siblings, and the proband resumed spontaneous puberty after discontinuation, with menarche occurring after recovery of gonadal function. Long-term care, however, remained dominated by obesity management and cardiometabolic surveillance. In both siblings, blood pressure remained within the normal range on repeated measurements; liver enzymes and hepatic ultrasonography were unremarkable with no evidence of NAFLD; and no current sleep-related symptoms or psychological/behavioral problems were reported (noting the younger brother’s remote history of OSA treated surgically). This documentation is important because structured assessment of obesity-related complications (including hypertension, NAFLD, OSA, and mental health concerns) is recommended in children with obesity. While sleep problems have been described in some *MC4R*-related case reports (26), neither sibling had current sleep-related symptoms.

Metabolic abnormalities still emerged over time. In the proband, metformin was introduced at 0.25 g three times daily as adjunctive therapy for impaired glucose tolerance and insulin resistance, together with intensified lifestyle intervention. She tolerated treatment well, with no significant adverse effects reported. However, because metformin exposure was brief and coincided with substantial weight reduction and stricter lifestyle measures, its independent contribution to the subsequent normalization of glucose tolerance cannot be determined. After body weight decreased and laboratory parameters improved, the parents requested discontinuation of metformin. As fasting hyperinsulinemia and HOMA-IR remained above the desirable range thereafter, we interpret the later metabolic profile more cautiously as residual insulin resistance despite improvement in glycemia and body weight, rather than complete resolution of insulin resistance.

Beyond the patients, the genetic diagnosis strengthened family risk awareness and engagement; notably, the father sought standardized adult care for his own glycemic and weight concerns, supporting the value of cascade counseling and coordinated long-term follow-up across generations.

Children are frequently referred to pediatric endocrinology because of rapidly progressive pubertal changes. When this presentation coexists with severe early-onset obesity (often beginning in the preschool years), accelerated linear growth with advanced bone age, disproportionate hyperinsulinemia/insulin resistance, or other early metabolic abnormalities, clinicians should consider an underlying genetic contribution to obesity and pursue etiologic evaluation. In our family, both siblings presented for evaluation of rapid pubertal progression, yet their marked weight gain beginning in early childhood, advanced bone age, and evolving metabolic risk prompted genetic testing. Whole-exome sequencing with segregation analysis identified a shared heterozygous *MC4R* c.494G>A (p. Arg165Gln) variant in both children, inherited from the father. This intrafamilial concordance suggests a meaningful genetic contribution and highlights that rapid pubertal tempo can be an important clinical “entry point” for recognizing monogenic obesity. Accordingly, when children present because of fast pubertal progression, clinicians should actively screen for red flags of genetic obesity (very early onset, severe phenotype, hyperphagia, and familial clustering) and consider genetic counseling/testing when appropriate.

This report has several limitations. First, as an observational case report involving only two siblings, it cannot establish a causal relationship between MC4R dysfunction and accelerated pubertal tempo. Second, in-house functional validation of the identified variant was not performed. Third, standardized instruments were not used to quantify lifestyle intervention or behavioral changes during follow-up; similarly, although no current sleep-related or psychosocial/behavioral concerns were reported, systematic screening with validated questionnaires was not undertaken. Fourth, final adult height outcomes are not yet available, particularly for the younger brother, which limits our ability to determine the ultimate auxological benefit of GnRHa therapy. Fifth, treatment was initiated relatively late, especially in the male sibling, and this may have attenuated any potential benefit on final height. Finally, some assessments and monitoring procedures were conducted in a real-world clinical setting rather than according to a strictly standardized research protocol. Despite these limitations, the dense longitudinal phenotyping before, during, and after GnRHa treatment, together with the intrafamilial concordance, provides clinically relevant lessons: in children presenting with rapidly progressive puberty plus severe early-onset obesity and metabolic risk, clinicians should consider monogenic obesity, evaluate for CNS pathology when central activation is identified in boys, and deliver integrated puberty and obesity management with sustained cardiometabolic surveillance.

## Conclusion

This report provides longitudinal data on Chinese siblings carrying the pathogenic *MC4R* c.494G>A (p. Arg165Gln) variant, illustrating the coexistence of severe early-onset obesity, accelerated pubertal tempo with central activation, and evolving metabolic comorbidities.

These cases underscore the need for integrated care in severe pediatric obesity: close monitoring of growth, bone age, and pubertal tempo; etiologic evaluation (including brain MRI in boys with central activation); judicious use of GnRHa when rapid progression threatens height potential; and sustained, guideline-informed surveillance and management of cardiometabolic complications with long-term lifestyle intervention and targeted adjunct pharmacotherapy when indicated.

## Data Availability

The original contributions presented in the study are included in the article/supplementary material, further inquiries can be directed to the corresponding author/s.

## References

[1] Loos R Yeo G . The genetics of obesity: from discovery to biology. Nat Rev Genet. (2022) 23:120–33. doi: 10.1038/s41576-021-00414-z. PMID: 34556834 PMC8459824

[2] Logan M Van der Merwe MT Dodgen TM Myburgh R Eloff A Alessandrini M . Allelic variants of the Melanocortin 4 receptor (MC4R) gene in a South African study group. Mol Genet Genomic Med. (2016) 4:68–76. doi: 10.1002/mgg3.180. PMID: 26788538 PMC4707032

[3] Hui H Yu Y Yiwei L Li Y Liling X Dongguang Z . Genetic etiology and clinical features of non-syndromic pediatric obesity in the Chinese population: a large cohort study. BMC Pediatr. (2025) 25:358. doi: 10.1186/s12887-025-05702-9. PMID: 40329189 PMC12057247

[4] Sridhar GR Gumpeny L . Melanocortin 4 receptor mutation in obesity. World J Exp Med. (2024) 14. doi: 10.5493/wjem.v14.i4.99239. PMID: 39713072 PMC11551707

[5] Wang P Yang S Zhou Q Zhang J Zhang Y Li D . Clinical and genetic analysis of a child with early-onset severe obesity. Zhonghua Yi Xue Yi Chuan Xue Za Zhi. (2023) 40:473–77. doi: 10.3760/cma.j.cn511374-20210411-00321. PMID: 36972945

[6] Liu P Tao J Ming M Zhang Z Lu G Chen W . A case of severe obesity complicated by severe pneumonia caused by a melanocortin-4 receptor gene mutation. Chin Pediatr Emergency Med. (2021) 28:1129–31. doi: 10.3760/cma.j.issn.1673-4912.2021.12.019. PMID: 41912385

[7] Dou X Gong C . A case of early-onset obesity with polycystic ovary syndrome caused by a heterozygous melanocortin-4 receptor variant. China Clin Case Results Database. (2022) 04:E7230. doi: 10.3760/cma.j.cmcr.2022.e07230. PMID: 41912385

[8] Huang X Yang W Liu Y Tang D Wu T Sun F . Mutations in MC4R facilitate the angiogenic activity in patients with orbital venous malformation. Exp Biol Med (Maywood NJ). (2020) 245:956–63. doi: 10.1177/1535370220919056. PMID: 32363922 PMC7427181

[9] Li H Ji CY Zong XN Zhang YQ . Height and weight standardized growth charts for Chinese children and adolescents aged 0 to 18 years. Zhonghua Er Ke Za Zhi. (2009) 47:487–92. doi: 10.3760/cma.j.issn.0578-1310.2009.07.003, PMID: 19951507

[10] Li H Ji CY Zong XN Zhang YQ . Body mass index growth curves for Chinese children and adolescents aged 0 to 18 years. Zhonghua Er Ke Za Zhi. (2009) 47:493–98. doi: 10.3760/cma.j.issn.0578-1310.2009.07.004, PMID: 19951508

[11] Yin J Li M Xu L Wang Y Cheng H Zhao X . Insulin resistance determined by Homeostasis Model Assessment (HOMA) and associations with metabolic syndrome among Chinese children and teenagers. Diabetol Metab Syndr. (2013) 5:71. doi: 10.1186/1758-5996-5-71. PMID: 24228769 PMC3833654

[12] Lavoie O Michael NJ Caron A . A critical update on the leptin‐melanocortin system. J Neurochem. (2023) 165:467–86. doi: 10.1111/jnc.15765. PMID: 36648204

[13] Farooqi IS Keogh JM Yeo GS Lank EJ Cheetham T O’Rahilly S . Clinical spectrum of obesity and mutations in the melanocortin 4 receptor gene. N Engl J Med. (2003) 348:1085–95. doi: 10.1056/NEJMoa022050. PMID: 12646665

[14] Thearle MS Muller YL Hanson RL Mullins M Abdussamad M Tran J . Greater impact of melanocortin-4 receptor deficiency on rates of growth and risk of type 2 diabetes during childhood compared with adulthood in Pima Indians. Diabetes. (2012) 61:250–57. doi: 10.2337/db11-0708. PMID: 22106157 PMC3237672

[15] Larsen LH Echwald SM Sørensen TIA Andersen T Wulff BS Pedersen O . Prevalence of mutations and functional analyses of melanocortin 4 receptor variants identified among 750 men with juvenile-onset obesity. J Clin Endocrinol Metab. (2005) 90:219–24. doi: 10.1210/jc.2004-0497. PMID: 15486053

[16] Krakoff J Ma L Kobes S Knowler WC Hanson RL Bogardus C . Lower metabolic rate in individuals heterozygous for either a frameshift or a functional missense MC4R variant. Diabetes. (2008) 57:3267–72. doi: 10.2337/db08-0577. PMID: 18835933 PMC2584132

[17] Nijenhuis WAJ Garner KM van Rozen RJ Adan RAH . Poor cell surface expression of human melanocortin-4 receptor mutations associated with obesity. J Biol Chem. (2003) 278:22939–45. doi: 10.1074/jbc.M211326200. PMID: 12690102

[18] Yeo GS Lank EJ Farooqi IS Keogh J Challis BG O’Rahilly S . Mutations in the human melanocortin-4 receptor gene associated with severe familial obesity disrupts receptor function through multiple molecular mechanisms. Hum Mol Genet. (2003) 12:561–74. doi: 10.1093/hmg/ddg057. PMID: 12588803

[19] Liu R Friedrich M Hemmen K Jansen K Adolfi MC Schartl M . Dimerization of melanocortin 4 receptor controls puberty onset and body size polymorphism. Front Endocrinol (Lausanne). (2023) 14:1267590. doi: 10.3389/fendo.2023.1267590. PMID: 38027153 PMC10667928

[20] Liu R Kinoshita M Adolfi MC Schartl M . Analysis of the role of the mc4r system in development, growth, and puberty of medaka. Front Endocrinol (Lausanne). (2019) 10:213. doi: 10.3389/fendo.2019.00213. PMID: 31024451 PMC6463759

[21] Mohsenipour R Rabbani A Amoli MM Asadi M Abbasi F . Relationship between a near Melanocortin-4 receptor gene variant and puberty timing in children is vague unlike obesity. J Diabetes Metab Disord. (2022) 21:1255–60. doi: 10.1007/s40200-022-01011-5. PMID: 36404836 PMC9672209

[22] Lam BYH Williamson A Finer S Day FR Tadross JA Gonçalves Soares A . MC3R links nutritional state to childhood growth and the timing of puberty. Nature. (2021) 599:436–41. doi: 10.1038/s41586-021-04088-9. PMID: 34732894 PMC8819628

[23] Sheffer-Babila S Sun Y Israel DD Liu S Neal-Perry G Chua SCJ . Agouti-related peptide plays a critical role in leptin’s effects on female puberty and reproduction. Am J Physiol Endocrinol Metab. (2013) 305:E1512–20. doi: 10.1152/ajpendo.00241.2013. PMID: 24169048 PMC3882375

[24] The Subspecialty Group of Endocrinologic HAMD Association CM The Editorial Board CJOP . Expert consensus on the diagnosis and treatment of central precocious puberty (2022). Zhonghua Er Ke Za Zhi. (2023) 60:507–515. doi: 10.3760/cma.j.cn112140-20220802-00693, PMID: 36594116

[25] Amodeo ME Deodati A Pedicelli S Mirra G Pampanini V Cianfarani S . Prevalence of organic central precocious puberty in males: criteria for a high index of suspicion. Endocr Connect. (2025) 14:e240405. doi: 10.1530/EC-24-0405. PMID: 39607418 PMC11728869

[26] Subspecialty Group of Endocrinologic HAMD . Expert consensus on diagnosis, assessment, and management of obesity in Chinese children. Zhonghua Er Ke Za Zhi. (2022) 60:507–15. doi: 10.3760/cma.j.cn112140-20220112-00043. PMID: 35658354

[27] Xue P Lin J Tang J Chen Y Yu T Chen C . Association of obesity and menarche SNPs and interaction with environmental factors on precocious puberty. Pediatr Res. (2024) 96:1076–83. doi: 10.1038/s41390-024-03168-6. PMID: 38649724

